# S194. INVESTIGATING PERIPHERAL MICRORNA-MRNA INTERACTIONS IN SCHIZOPHRENIA

**DOI:** 10.1093/schbul/sby018.981

**Published:** 2018-04-01

**Authors:** Michael Geaghan, Murray Cairns

**Affiliations:** University of Newcastle

## Abstract

**Background:**

Schizophrenia is a severe neuropsychiatric disorder, characterised by positive and negative symptoms, and cognitive deficits. High throughput technologies such as microarrays, and more recently next-generation sequencing have identified numerous genetic variants and transcriptional signatures associated with schizophrenia. Over the last decade, microRNAs (miRNAs) have been found differentially expressed in both peripheral and post-mortem grey matter tissue in schizophrenia, and three genome-wide significant schizophrenia-associated variants occur within two miRNA loci – MIR137 and MIR548AJ2. These small, non-coding RNAs are potent regulators of translation, can target a wide variety of transcripts, and are therefore of particular interest in polygenic disorders such as schizophrenia.

**Methods:**

We obtained total RNA isolated from peripheral blood mononuclear cell (PBMC) samples from the Australian Schizophrenia Research Bank (ASRB). The samples included 36 individuals with schizophrenia and 15 healthy controls. We utilised small RNA and mRNA sequencing technology to examine both the miRNA and mRNA expression profiles of these sample. Raw reads were aligned to the human genome (hg38), annotated, counted and analysed for differential expression using an open source software pipeline. Correlations between miRNA and mRNA expression were found and matched to predicted TargetScan miRNA-mRNA interactions using the miRComb R package. Ingenuity Pathway Analysis and Gene Set Enrichment Analysis were performed to identify pathways and gene ontologies enriched for differentially expressed genes.

**Results:**

35 miRNAs and 97 genes were differentially expressed (FDR<0.1); most miRNAs (21 out of 35) were downregulated, while the vast majority of mRNAs (80 out of 97) were upregulated. When males and females were analysed separately, we found 14 miRNAs and 365 genes differentially expressed in males, while females only showed 7 miRNAs and 1 gene (NRCAM – neuronal cell adhesion molecule) differentially expressed. Several miRNAs in males were found to significantly correlate with differentially expressed genes, including miR-1271-5p with schizophrenia candidate gene DGCR2. Furthermore, many differentially expressed genes and miRNAs have previously been linked to schizophrenia and neuronal function. Among males, several immune- and inflammation pathways were enriched for differentially expressed genes. Interestingly, while upregulated genes were enriched for immune-related gene ontologies, downregulated genes were enriched for gene ontologies relating to development.

**Discussion:**

These results contribute to a growing body of evidence that suggest peripheral miRNA and mRNA expression is altered in schizophrenia. We identify a general downregulation of miRNAs and upregulation of mRNAs in peripheral tissue in schizophrenia. Several significant correlations between miRNAs and mRNAs previously linked to schizophrenia and brain function suggest potential miRNA-mRNA interactions that may be significant for disease pathophysiology.

